# Case Report: Evolution and targeted therapy of an EGFR-mutant large-cell neuroendocrine carcinoma

**DOI:** 10.3389/fonc.2025.1599765

**Published:** 2025-10-27

**Authors:** Li Jiang, Xiaowen Yao, Xiuyu Cai, Pengfei Li

**Affiliations:** ^1^ Department of Traditional Chinese Medicine, Sun Yat-Sen University Cancer Center, State Key Laboratory of Oncology in South China, Collaborative Innovation Center for Cancer Medicine, Guangzhou, China; ^2^ Department of Traditional Chinese Medicine, Southern Medical University Hospital of Integrated Traditional Chinese and Western Medicine, Guangzhou, China

**Keywords:** large-cell neuroendocrine carcinoma (LCNEC), EGFR, EGFR exon 21 L858R, TKI (tyrosine kinase inhibitor), lung cancer

## Abstract

We report the case of a 47-year-old female non-smoker diagnosed with stage IV large-cell neuroendocrine carcinoma (LCNEC) of the lung harboring an EGFR exon 21 L858R mutation. The patient exhibited a sustained response to first-line osimertinib, with a progression-free survival of 20 months, followed by transformation to small-cell lung cancer (SCLC) confirmed via histopathological reassessment. Second-line treatment with etoposide and cisplatin combined with radiotherapy resulted in an additional 7 months of disease control. Subsequent progression was accompanied by features suggestive of adenocarcinoma, supported by elevated carcinoembryonic antigen levels, stable neuron-specific enolase, and circulating tumor DNA profiling. Third-line chemotherapy with paclitaxel, carboplatin, and bevacizumab, followed by maintenance therapy with aumolertinib and anlotinib, extended progression-free survival by 21 months. Overall survival reached 48 months. This case highlights the critical importance of repeated molecular profiling and histologic reevaluation in guiding therapeutic decisions for EGFR-mutant LCNEC undergoing phenotypic evolution.

## Introduction

Large-cell neuroendocrine carcinoma represents a rare and aggressive subtype of non-small cell lung cancer, comprising approximately 3% of all pulmonary malignancies (1). It is characterized by poor prognosis and limited therapeutic options (2). While EGFR mutations are seldom observed in pure LCNEC (estimated frequency <5%), they occur with greater frequency in mixed forms exhibiting adenocarcinomatous differentiation (3). Existing literature suggests that EGFR-tyrosine kinase inhibitors may confer benefit in this molecularly selected subgroup, although acquired resistance frequently emerges through mechanisms such as histologic transformation (4). The molecular mechanisms underlying such transformations, including potential roles of RB1/TP53 co-mutations and neuroendocrine marker dynamics, remain incompletely understood (5). Herein, we present a detailed case of an EGFR-mutant LCNEC that underwent sequential morphologic evolution into SCLC and subsequently displayed characteristics consistent with adenocarcinoma, underscoring the dynamic nature of therapeutic resistance and the value of adaptive treatment strategies (6).

## Case presentation

A 47-year-old female with no smoking history presented in November 2018 with an intermittent cough of three weeks’ duration. Physical examination revealed an Eastern Cooperative Oncology Group (ECOG) performance status of 1. Contrast-enhanced computed tomography (CT) of the chest showed a heterogeneously enhancing mass measuring 41 mm × 42 mm in the right upper lobe. Brain magnetic resonance imaging (MRI) revealed a solitary 15 mm × 15 mm enhancing lesion within the right cerebellar hemisphere. Histopathological examination of transbronchial biopsy specimens revealed nests and trabeculae of large polygonal cells with abundant cytoplasm, coarse chromatin, and frequent mitoses (15 per 2 mm²) (2). Immunohistochemistry showed strong positivity for synaptophysin, chromogranin A, and CD56, Molecular profiling identified an EGFR exon 21 L858R mutation (7). Thus, the diagnosis was large cell cancer in the upper lobe of the right lung with solitary brain metastasis (cT2N2M1 stage IV with EGFR exon 21 L858R mutation).

**Figure 1:**
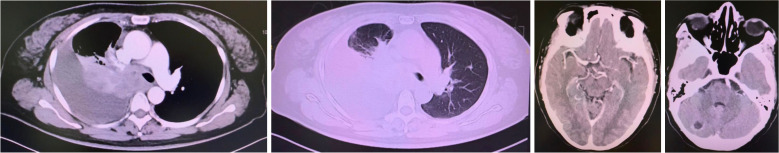
Progression after Osimertinib from November 2018 to June 2020. Small cell transformation confirmed by rebiopsy.

First-line therapy with osimertinib 80 mg once daily was initiated (4). After two months, restaging CT showed partial response with reduction in the lung mass to 28 mm × 25 mm. Follow-up brain MRI confirmed complete resolution of the cerebellar metastasis. The patient remained progression-free for 20 months until June 2020 when repeat imaging demonstrated disease progression (8). (**Figure 1**).

**Figure 2:**
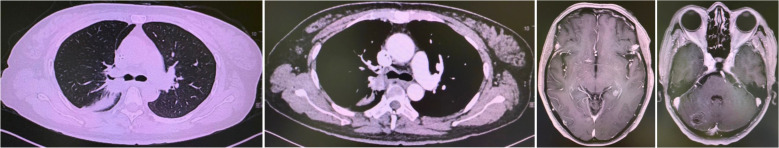
Partial response after etoposide and cisplatin #4.

**Figure 3:**
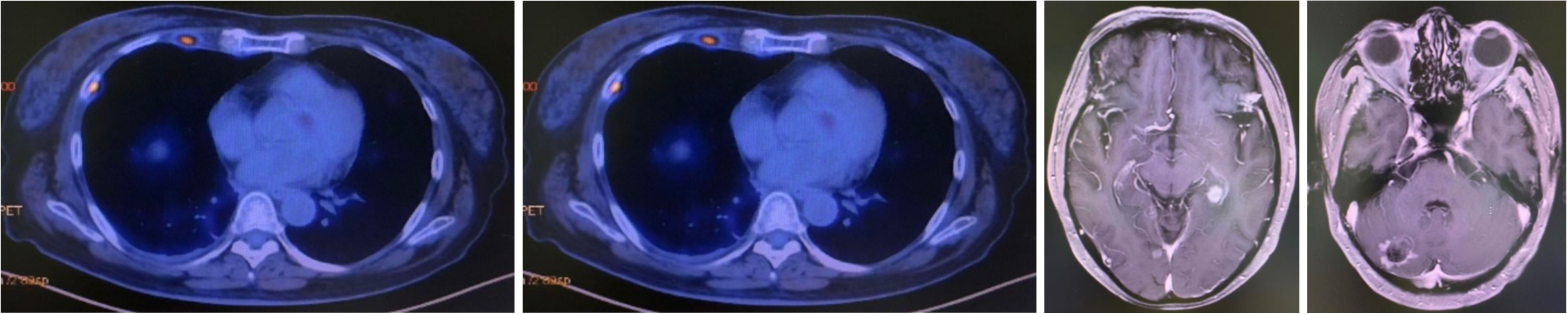
Progression after chemotherapy holiday since October 2020.

**Figure 4:**
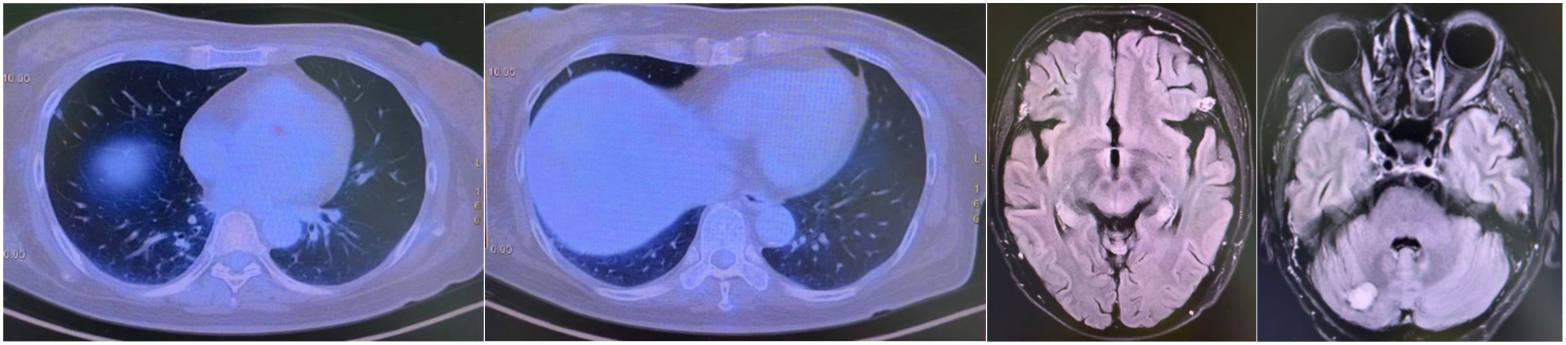
Partial response after paclitaxel, carboplatin, and bevacizumab #4.

**Figure 5:**
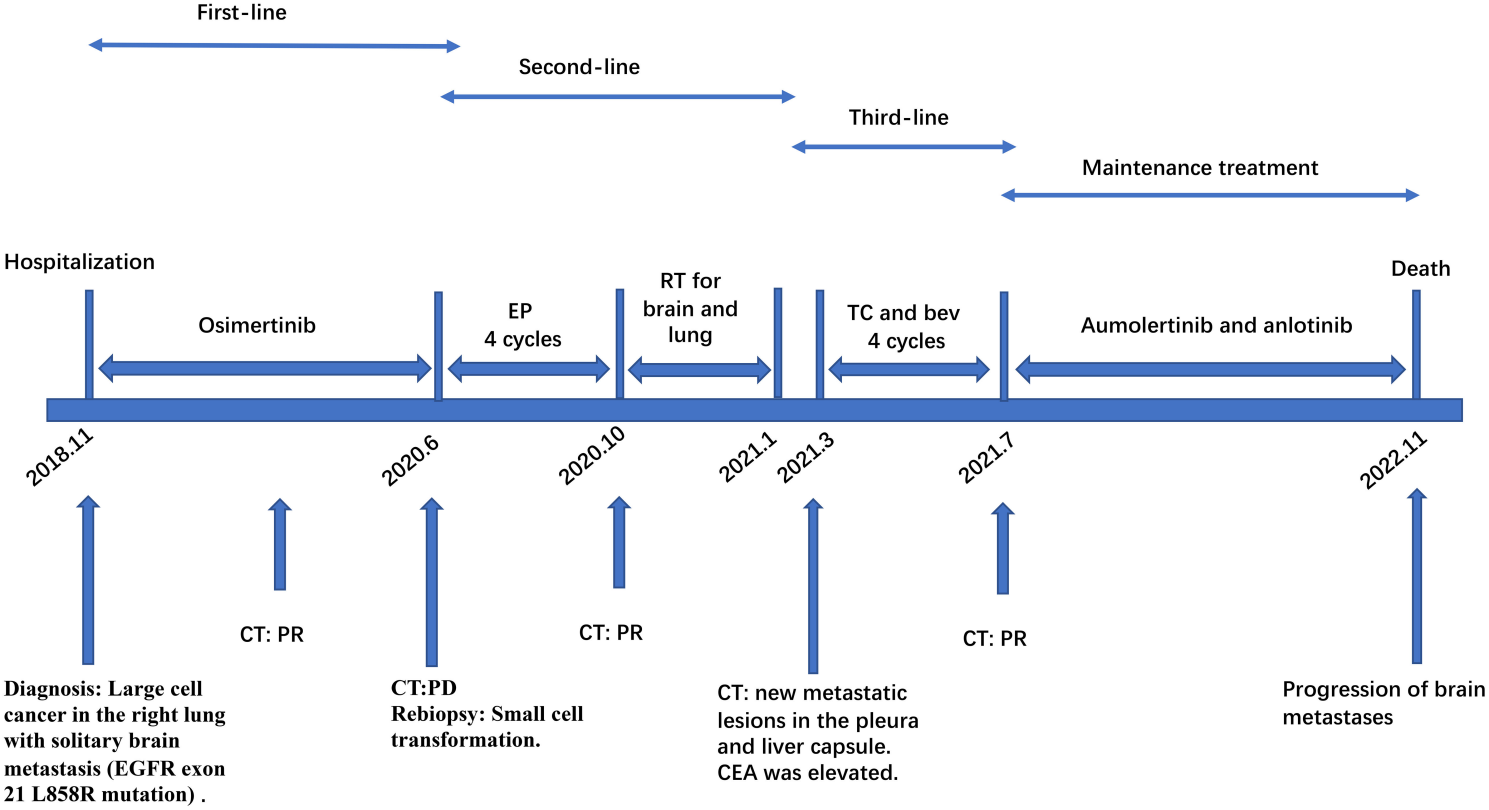
Multi-line TKIs therapy schedule. EP, etoposide combined with cisplatin; TC and bev, paclitaxel, carboplatin, and bevacizumab; PR, partial response; PD, progressive disease; SD, stable disease; TKIs, tyrosine kinase inhibitors.

A CT-guided biopsy revealed transformation to small-cell lung cancer with maintained neuroendocrine marker expression. Second-line therapy with etoposide (100 mg/m² days 1-3) and cisplatin (75 mg/m² day 1) was administered for four cycles (9). CT scans showed that the lung lesions had significantly shrunk. (**Figure 2**) The patient subsequently received consolidative radiotherapy: whole-brain radiotherapy (39 Gy in 13 fractions) followed by intensity-modulated radiotherapy to the primary lung tumor (45 Gy in 30 fractions) (10, 11). Histological analysis from a re-biopsy of the progressive chest lesion revealed small cell cancer transformation and tested positive for neuroendocrine markers, including synaptophysin, chromogranin A, and CD56. Tissue and blood samples were subjected to next-generation sequencing (NGS), which revealed an EGFR exon 21 L858R deletion. In March 2021, PET/CT and brain MRI revealed new metastatic lesions in the pleura and liver capsule, with an increase in multiple metastatic tumors in the brain. (**Figure 3**) Serum tumor markers showed elevated carcinoembryonic antigen (CEA) (8.1 ng/mL) while NSE remained normal (11.3 ng/mL). Following disease progression, a third biopsy of the chest mass was performed, but no tumor tissue was detected. Circulating tumor DNA (ctDNA) from peripheral blood revealed an EGFR exon 21 L858R deletion. We considered that the recurrent focus might be adenocarcinoma of the lung. Plasma ctDNA analysis confirmed persistence of EGFR L858R mutation without additional resistance alterations. Third-line therapy (from April 20 to July 21, 2021) with paclitaxel (200 mg/m²), carboplatin (AUC 5), and bevacizumab (15 mg/kg) was administered every three weeks (12). After four cycles, CEA decreased to 3.9 ng/mL, and CT imaging showed a partial response (**Figure 4**). Treatment was complicated by grade 2 epistaxis, leading to bevacizumab discontinuation. The patient was switched to maintenance therapy with aumolertinib (110 mg daily) combined with anlotinib (12 mg daily, days 1–14 every 21 days) (13, 14). Follow-up assessments at 2, 6, 10, and 14 months showed sustained clinical and radiographic stability. The patient ultimately died due to progressive brain metastases in November 2022, with an overall survival of 48 months. The course of treatment is presented in **Figure 5**.

## Discussion

This report illustrates the clinical course of a patient with EGFR-mutant LCNEC who experienced multiple histologic transformations throughout her treatment (15). The presence of an EGFR sensitizing mutation in LCNEC is an uncommon finding, and its therapeutic implications remain incompletely defined (16). In this instance, initial response to osimertinib was robust but finite, ultimately limited by transformation to a small-cell phenotype—an established mechanism of resistance in EGFR-driven lung cancers (17, 18). Given the common genetic and clinical factors between LCNEC and SCLC, we believe that histological transformation to LCNEC can be a mechanism of acquired EGFR-TKI resistance. Rebiopsy is recommended when EGFR-TKI resistance is detected, especially in rapidly progressing or highly invasive lesions. In this case, the patient initially responded well to the third-generation EGFR-TKI and achieved a PFS of 20 months, consistent with the FLAURA study (19).

The emergence of SCLC was confirmed via immunohistochemical and morphologic reevaluation (3). The patient’s response to platinum-etoposide chemotherapy aligns with existing knowledge regarding the chemosensitivity of SCLC (20). Of particular interest, her subsequent progression exhibited biologic features more consistent with adenocarcinoma, as reflected by tumor marker trends and ctDNA profiles (21). This observation suggests either a second transformation or the outgrowth of a pre-existing adenocarcinomatous clone (6).

This case highlights the crucial role of tumor microenvironment (TME) dynamics in therapeutic responses. Recent pan-cancer studies reveal that molecular alterations like PLIN3/EPHB2 dysregulation and hypoxia-related signatures consistently promote M2 macrophage infiltration and immunosuppression, correlating with adverse outcomes across malignancies (22). These TME modifications likely contributed to both innate and acquired resistance in this EGFR-mutant LCNEC, particularly through immunosuppressive macrophage recruitment and hypoxia-mediated pathways (23). The studies’ integrated multi-omics methodologies—combining bulk/single-cell transcriptomics, spatial profiling, and computational algorithms—provide a framework for evaluating TME immune composition. Importantly, identified compounds (e.g., clofibrate targeting PLIN3) suggest actionable strategies for modulating the TME in neuroendocrine carcinomas. This underscores the need for combined targeting of oncogenic drivers and microenvironmental factors in treatment-resistant cases (24, 25).

The serial application of molecular and pathologic diagnostics was instrumental in guiding therapeutic decisions at each juncture (16). The persistent detection of the EGFR L858R mutation supported the rechallenge with EGFR-directed therapy in combination with antiangiogenic treatment, which may have contributed to the prolonged survival observed (12).

Radiotherapy details have been explicitly provided, with dose and fractionation specified for both brain and lung treatments (26, 27). These consolidative approaches likely contributed to the prolonged survival observed, particularly in the oligometastatic setting (28).

From a tumor microenvironment perspective, while immunotherapy was not employed in this case, emerging evidence suggests that neuroendocrine tumors may exhibit distinct immune profiles characterized by altered cytokine expression and immune cell infiltration patterns. These finding provide valuable insights into how TME components may influence treatment response in related malignancies (29).

This case underscores the critical importance of repeated molecular and histologic assessment in guiding therapy for EGFR-mutant LCNEC (30). Future research should focus on optimizing treatment sequencing, developing predictive biomarkers for histologic transformation, and exploring novel therapeutic approaches including combination strategies targeting both EGFR and neuroendocrine pathways.
